# Effect of Coffee Silverskin on Meat Quality of Growing Rabbits

**DOI:** 10.3390/foods14050812

**Published:** 2025-02-26

**Authors:** Francesco Foti, Manuel Scerra, Pasquale Caparra, Matteo Bognanno, Caterina Cilione, Paolo Fortugno, Paolo De Caria, Valerio Chinè, Guido Mangione, Salvatore Gagliano, Luigi Chies

**Affiliations:** 1 Department of Agriculture, Animal Production, University of Reggio Calabria, Via dell’Università, 25, 89124 Reggio Calabria, Italy; francesco.foti@unirc.it (F.F.); pasquale.caparra@unirc.it (P.C.); matteo.bognanno@unirc.it (M.B.); caterina.cilione@unirc.it (C.C.); frtpla99s23h224h@studenti.unirc.it (P.F.); paolo.decaria@unirc.it (P.D.C.); lchies@unirc.it (L.C.); 2 Caffè Mauro SpA, Zona Industriale Snc, 89018 Villa San Giovanni, Italy; 3 Department of Agriculture, Alimentazione e Ambiente (Di3A), University of Catania, Via Santa Sofia 100, 95123 Catania, Italy; guido.mangione@unict.it (G.M.); salvator.gagliano@tiscali.it (S.G.)

**Keywords:** polyphenols, shelf-life, by-products, fatty acid

## Abstract

The aim of the present study was to assess the impacts of coffee silverskin (CSS) inclusion in rabbit diets in regard to meat quality. A total of 30 Hycole rabbits were divided into two groups of 15 animals per group and fed with a basal diet (C group) or with the same basal diet but with 10% of CSS (CSS10 group) as a partial cereal replacement. Integration of 10% CSS in the rabbit diet increased dry matter intake (DMI, *p* < 0.05) and tended to increase (*p* = 0.096) the final body weight. The CSS diet tended to reduce the concentration of C18:3 ω-3 (*p* = 0.089), C20:5 ω-3 (*p* = 0.064) and C22:5 ω-3 (*p* = 0.069) in muscle compared to the control diet, negatively affecting the ω-6/ω-3 ratio (*p* < 0.05), which was higher in the CSS10 group compared to the control group. Finally, the addition of CSS to the rabbit diet made the meat more resistant (*p* < 0.01) to lipid oxidation. Further research is needed to better understand the reasons for improved oxidative stability in meat following dietary CSS supplementation.

## 1. Introduction

The latest UN projections suggest that the world population could grow to around 8.5 billion in 2030 and 9.7 billion in 2050, reaching a peak at around 10.4 billion people during the 2080s and remain at that level until 2100 [1]. Considering these data, it is presumable that food production will also increase, leading to a further increase in biomass from the processing of agricultural products. These wastes can cause environmental problems as well as having a huge impact on industrial economic costs for disposal and regeneration. Never before has it been necessary to valorize these biomasses to improve circular economy models, reducing the accumulation of these wastes and transforming them into biodegradable and useful materials. Furthermore, the reuse of agro-industrial residues could increase the economic and added value of materials, products that could be used in various other sectors, such as in food packaging, as fertilizers and biomaterials, or as animal feed [2,3,4,5].

Among the most widely produced commodities is coffee, with a global production of almost 10 million tons [6], from which, during industrial processing, approximately 90% is discarded [7]. Among these wastes, a stand out is coffee silverskin (CSS), the external layer of the peel that comes entirely off during the roasting phase of the green coffee bean, a by-product representing around 4% of the bean [8]. CSS is a by-product that is relatively stable compared to other coffee by-products due to its low moisture content [9]. Various health benefits have been attributed to this by-product [10], linked, above all, to the high quantity of antioxidant compounds such as polyphenols [11]. In fact, the high levels of antioxidant activity in CSS are mainly linked to the high concentration of polyphenols in coffee beans as well as the compounds generated by the Maillard reaction, such as melanoidins [12]. Furthermore, CSS is rich in protein, and the presence of fiber and ash in high quantities suggests a significant mineral content [13]. However, some factors could negatively influence the reuse of CSS in the food industry, such as caffeine and acrylamide content. However, the latter have always been detected in roasted coffee (from which silverskin was collected) well below the limits allowed by current European legislation [14]. Regarding caffeine, data from some studies on monogastric animals [15,16] showed low concentrations of caffeine, lower than those reported for coffee drinks, with 10% supplementation of coffee pulp in the diets. Furthermore, Tsigkou et al. [17] emphasized that its caffeine levels and the presence of bioactive compounds have led to CSS being considered as a safe nutraceutical.

In the literature, there are many studies evaluating coffee by-products other than CSS, such as coffee pulp or coffee husks, in the diets of cattle [18,19], sheep [20,21,22,23,24,25], pigs [26,27] and chickens [28,29,30]; however, to the best our knowledge, no study has investigated the effects of CSS supplementation in the diets of small monogastric animals, such as rabbits, on meat quality. Therefore, the aim of the present study was to assess the impacts of 10% DM CSS inclusion in rabbit diets on meat quality. We mainly hypothesized an improvement in the oxidative stability of rabbit meat by supplementing CSS in their diet.

## 2. Materials and Methods

### 2.1. Animals and Diet

The Animal Welfare Committee of the University of Reggio Calabria approved this experimental trial (prot. No. 1214).

The experiment lasted 8 weeks and was conducted with 30 Hycole rabbits which were 4 weeks old, with a mean weight of 638.5 ± 2.07 g, housed individually in wire cages. They were randomly divided into two groups of 15 animals per group and fed with a basal diet (C group) or with the same basal diet in which part of cereals was replaced with 10% coffee silverskin (CSS10 group). The chemical composition of the two experimental diets was (g/kg DM) DM 901 (g/Kg wet weight), crude protein 157, ether extract 23.8, ash 35.9, NDF 330, total extractable phenols 4.30 (g of tannic acid equivalent/Kg DM) and α-Tocopherol 52.4 (μg/g DM) for the control diet; DM 908 (g/Kg wet weight), crude protein 164, ether extract 28.2, ash 33.3, NDF 345, total extractable phenols 5.94 (g of tannic acid equivalent/Kg DM) and α-Tocopherol 51.9 (μg/g DM) for the CSS10 diet (Table 1).

Rabbits were adapted for 7 days to a respective experimental diet. Coffee silverskin was obtained from a 50:50 mixture of *Coffea arabica* (Arabica) and *Coffea canephora* (Robusta), provided by Caffè Mauro S.P.A. (Villa San Giovanni, RC, Italy). Through the feeders in the cages, diets were supplied ad libitum (pellet form) with free access to water. Animals were weighed every 10 days and feed consumption was evaluated every day.

At the end of the experiment, all the rabbits were weighed for the last time, slaughtered (fasting for 4 h) and eviscerated to calculate the carcass weight. Carcasses were subsequently chilled at +4 °C for 24 h. The *longissimus thoracis et lumborum* (LTL) muscle was used for chemical analyses. The fore part of the LTL muscle was used to evaluate oxidative stability in raw meat, while the remaining parts of the muscle were stored at −20 °C and subsequently used for analyses of the proximate composition, fatty acid profile, and antioxidant vitamins of meat.

### 2.2. Feeds Chemical Analyses

Following the methods described by AOAC [31], the ether extract (method 920.39), dry matter (method 934.01), crude protein (method 984.13) and ash contents (method 942.05) of experimental feed samples were quantified. Neutral detergent fiber (NDF) was determined as described by Van Soest et al. [32], while the fatty acid composition of the experimental diets was determined following the procedures described by Gray et al. [33]. The Folin–Ciocalteau method, modified by Luciano et al. [34], was used to determine total extractable phenols. The method described by Rufino-Moya et al. [35] was followed to evaluate tocopherols from 200 mg of feed samples. Acrylamide was determined according to the standard methodology EN 16618:2015, with liquid chromatography combined with tandem mass spectrometry [36].

### 2.3. Meat Quality Analysis

The proximate analyses of meat were carried out following the methods proposed by the Association of Official Analytical Chemists [31].

The fatty acid profile of meat was measured following the procedures described by Folch et al. [37]. Briefly, intramuscular fat was extracted from 5 g of muscle using a 2:1 (*v*:*v*) chloroform–methanol solution. Subsequently, a 100 mg portion was methylated by adding 0.05 mL of 2 N methanolic potassium hydroxide and 1 mL of hexane [38], with nonanoic acid acting as an internal standard (Sigma-Aldrich, St. Louis, MO, USA). A ThermoQuest gas chromatograph (GC) was used for the analyses (ThermoQuest, Milan, Italy, with a 100 m high-polar fused silica column, i.d. 0.25 mm, film thickness 0.25 μm). The condition of GC and FAME identification was performed as reported by Scerra et al. [4]. Atherogenic and thrombogenic indexes were calculated following the formulas indicated by Ulbricht and Southgate [39]. Meat samples were analyzed to evaluate cholesterol and antioxidant vitamins using the method reported by Natalello et al. [40] with a UHPLC system (Shimadzu Corporation, Kyoto, Japan).

Thiobarbituric acid reactive substances (TBARSs) were determined to evaluate lipid oxidation on meat using three slices (2 cm thick) of meat, covered with PVC film, stored at 4 °C in the dark for 2 h (day 0), 3 and 7 days. A TBARS assay was evaluated for each meat sample on each day of storage [41]. Each monitoring day, 2.5 g of meat was homogenized with distilled water (12.5 mL) for 2 min. Subsequently, trichloroacetic acid (12.5 mL, 10% *w*/*v*) was added, vigorously vortexed, and filtered (Whatman No. 1 filter paper, Buckinghamshire, UK). From the filtrate, 4 mL was taken and combined with 1 mL of 0.06 M aqueous thiobarbituric acid and incubated in a water bath (80 °C for 90 min). Using solutions of known concentrations of 1,1,3,3,-tetra-ethoxypropane in distilled water, the assay was calibrated, covering the concentrate range of 5 to 65 nmoles/4 mL. Results were expressed as mg of malonaldehyde (MDA)/kg of meat. Using a UV-1800 Shimadzu spectrophotometer, the absorbance was measured at 532 nm (Shimadzu Corporation, Milan, Italy).

### 2.4. Statistical Analysis

The software Minitab 19 (Minitab Inc., State College, PA, USA) was used to analyze all the data (dietary treatments as factors in ANOVA analysis and considering single animals as statistical unit). The effect of the experimental diet on proximate composition, fatty acid composition and animal performance was analyzed using a one-way ANOVA, while a mixed model for repeat measures was used to analyze the data of the TBARS assay, where the terms in the model were dietary treatment, time of refrigerated storage and their interaction as a fixed factor. Individual animals acted as a random factor.

Using Tukey’s multiple comparison test, differences between means were assessed. Significance was declared at *p* ≤ 0.05, whereas trends were considered when 0.05 < *p* ≤ 0.10.

## 3. Results and Discussion

In this study, the use of coffee silverskin in rabbit diets was investigated for the first time. CSS, being a by-product derived from a roasting process, could contain compounds that are formed during this process, such as acrylamide, some of which are considered potentially dangerous for humans [42]. In the European Union, the commission regulation (EU) 2017/2158 indicated the threshold levels for acrylamide content in food products, establishing a level of 400 µg/kg for roasted coffee. The CSS used in this trial had a value of 141 µg/kg (provided by Caffè Mauro S.p.a.), below the guideline values. Consequently, 17.5 g of CSS, the average amount of CSS ingested daily by CSS rabbits, would contain approximately 2.5 µg of acrylamide. The amount of acrylamide found in the CSS used in this study is similar to those found by other authors [43].

In this trial, CSS supplementation in the rabbit diet influenced the main growth performance parameters (Table 2). Integration of 10% CSS in the rabbit diet tended to increase (*p* = 0.096) the final body weight. In the CSS10 group, a higher DMI (*p* < 0.05) was observed compared to the C group, indicating a higher amount of feed ingested (175 vs. 154 g/d for the CSS10 and C groups, respectively) by the animals that received CSS supplementation. The higher DMI certainly influenced the final body weight that tended to be higher in the rabbits of the CSS10 group compared to the control rabbits.

In the literature, there are many studies that have evaluated coffee by-products in the diets of cattle [18,19], sheep [20,21,22,23,24,25], pigs [26,27] and chickens [28,29,30], but there are no studies on rabbits. Furthermore, all the experimental trials related to the studies indicated above were conducted using other coffee by-products such as coffee pulp or coffee husks. Carvalho et al. [27] did not observe differences in the feed intake, weight gain, feed conversion and carcasses of pigs fed diets containing 0, 2, 8, 12 and 16% of ensiled coffee pulp. Also, when Funes et al. [27] tested the inclusion of dehydrated coffee pulp in the diets of male chickens, they did not observe differences in consumption, weight gain and feed conversion at inclusion levels of up to 20% (in the fattening stage). CSS is characterized by the highest lipid content among all the coffee by-products [17], increasing fat levels in the CSS diet (28.2 vs. 23.8 g/kg DM in CSS and C diets, respectively). Presumably, the increased fat levels in the CSS diet may have encouraged feed consumption. This higher feed consumption in CSS rabbits compared to C rabbits influenced positively final body weight. In addition, CSS is also characterized by a high protein content, higher than other coffee by-products [17], leading to slightly higher protein levels in the CSS diet compared to the C diet (164 vs. 157 g/kg DM, respectively).

CSS integration did not affect the chemical composition of the meat (Table 2). In fact, the values of crude protein, moisture, ether extract, and ash in meat were comparable between experimental groups.

The effects of CSS administration on individual meat FAs are shown in Table 3. Partial replacement of cereals with 10%CSS did not lead to any changes in IMF accumulation (*p* = 0.141) and the fatty acid composition of meat. The total of saturated fatty acids (SFAs), monounsaturated fatty acids (MUFAs) and polyunsaturated fatty acids (PUFAs) were not different between groups. However, the CSS diet tended to reduce the concentration of linolenic acid (C18:3 ω-3, *p* = 0.089), eicosapentaenoic acid (EPA, C20:5 ω-3; *p* = 0.064) and docosapentaenoic acid (DPA, C22:5 ω-3; *p* = 0.069) in muscle compared to the control diet.

These data affected the total ω-3 Fas, which tended to decrease (*p* = 0.091) in the meat of rabbits receiving CSS supplementation compared to the meat from control rabbits, negatively affecting the ω-6/ω-3 ratio (*p* < 0.05), which was higher in the CSS10 group compared to the control group. The decrease in the level of ω-3 fatty acids in the meat of the rabbits from the CSS10 group was mainly influenced by the characteristics of the diet. In fact, CSS supplementation in the diet led to a decrease in the level of C18:3 ω-3. Other authors [9,42] have also found low levels of C18:3 n-3 in CSS. The lower levels of ω-3 PUFAs negatively influenced the thrombogenic index, which was higher (*p* < 0.05) in meat from the CSS10 group than in the control meat, as well as the atherogenic index (*p* < 0.05).

The main process that most degrades meat during storage is lipid oxidation, with unsaturated fatty acids being more subject to this degradation process [44], a phenomenon that inevitably leads to a deterioration of the organoleptic qualities of meat. Consequently, finding feeding strategies that maintain meat safety and sensory acceptability is of fundamental importance. The diet provided to the animal is among the factors that mostly affect oxidative stability, influencing the content of PUFAs and antioxidant compounds in meat. Antioxidants play a fundamental role during meat storage [5], protecting compounds such as PUFAs from oxidative processes [45].

**Figure 1:**
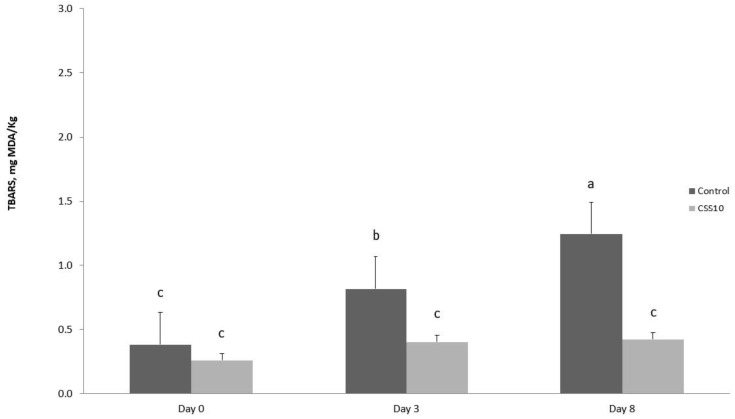
Coffee silverskin supplementation on lipid oxidation (TBARS assay) in meat over aerobic storage (at 4 °C). Control, basal diet; GS, basal diet supplemented with 10% coffee silverskin. ^a,b,c^ Values with different superscripts are significantly different (*p* < 0.05).

In this study, the addition of 10% CSS to the rabbit diet made the meat more resistant to lipid oxidation (Figure 1). CSS supplementation in the rabbit diet reduced TBARS values (*p* < 0.01) and protected meat from lipid oxidation over time (*p* < 0.01). The diet × time interaction was significant (*p* < 0.01). While a linear increase over time (*p* < 0.01) of lipid oxidation was observed in C meat, in the CSS meat, the TBARS values were comparable for all observation days, indicating that the malondialdehyde content in the CSS meat did not change due to the lower lipid oxidation.

One of the main molecules that plays an important antioxidant action in meat is α-tocopherol [46], a fat-soluble vitamin that protects fatty acids from oxidation. However, feeding a diet with 10% CSS had no effect (*p* > 0.10) on the content of α-tocopherol in rabbit meat (Table 2). There is no information on the effect of CSS on the tocopherol content of rabbit meat. Different studies [9,47,48] report high antioxidant activity for CSS. Jiménez-Zamora et al. [48] show a value of 598 mmol trolox/g DM of CSS regarding the antiradical activity against ABTS. Chemat et al. [49] state that the antioxidant properties of coffee silverskin are derived from its polyphenolic content. Some researchers [50] have hypothesized a protective effect at the intestinal level of phenolic compounds toward vitamin E and other antioxidants, allowing for the absorption of a greater amount of these molecules, leading to a greater accumulation in the tissues. However, our results do not confirm this hypothesis, similarly to the work of Gessner et al., [51] who studied pigs whose diets were supplemented with grape tannins. Further research is needed to better understand this phenomenon.

## 4. Conclusions

The findings of the present study suggest that adding 10% CSS to the diet of growing rabbits can improve the final body weight of the animals, and this may be influenced by the higher DMI of the rabbits in the CSS group compared to the control rabbits. Partial replacement of cereals with 10% CSS tended to reduce the concentration of C18:3 ω-3, C20:5 ω-3, and C22:5 ω-3 in muscle, negatively affecting the ω-6/ω-3 ratio, which was higher in the CSS10 group compared to the control group, while enhancing meat oxidative stability. Some studies have hypothesized a protective effect at the intestinal level of polyphenolic toward vitamin E, one of the main molecules that is involved in important antioxidant action in meat, allowing for the absorption of a greater amount of these molecules. However, feeding a diet with 10% CSS had no effect on the content of α-tocopherol in rabbit meat. Further studies are needed to better understand this phenomenon, focus on optimizing the CSS inclusion levels, and evaluate its long-term effects on meat quality and consumer acceptance.

## Figures and Tables

**Table 1:** Ingredients (% on DM basis) and chemical composition of the experimental diets.

	C Diet	CSS10 Diet	CSS
Barley	10	5	
Maize	10	5	
Wheat bran	28	28	
Soybean meal	10	10	
Alfalfa meal	40	40	
Coffee silverskin	-	10	
Vitamin mineral premix ^1^	2	2	
Chemical composition			
Dry matter (DM) g/kg wet weight	901	908	931
Crude protein g/kg DM	157	164	190.1
Ether extract g/kg DM	23.8	28.2	15.2
Ash g/Kg DM	35.9	33.3	80.1
NDF g/Kg DM	330	345	600
Total extractable phenols (g TAe ^2^/kg DM)	4.30	5.94	10.2
α-Tocopherol (μg/g *DM)*	52.4	50.2	30.1
Fatty acids (g/100 g of total fatty acid)			
C10:0	0.01	0.04	0.01
C12:0	0.04	0.06	0.01
C14:0	0.13	0.21	0.90
C16:0	15.8	15.9	18.7
C18:0	4.15	4.2	5.30
C18:1 *n*-9	36.8	31.2	8.40
C18:2 *n*-6	25.8	25.2	22.4
C18:3 *n*-3	1.89	1.61	0.49

^1^ The mineral vitamin premix consisted of vitamin A 6750 UI; vitamin D3 1000 UI; vitamin E 2 mg; vitamin B12 0.01 mg; vitamin B1 1 mg; folic acid 0.2 mg; D-pantotenic acid 5 mg; Co 0.05 mg; Mn 12.5 mg; Zn 15 mg; Mo 0.5 mg; ^2^ tannic acid equivalent.

**Table 2:** Rabbit performances in vivo and in the chemical composition of muscle (g/100 g wet weight).

	Dietary Treatment ^1^		SEM ^6^	*p* Value
	C	CSS10		
Final BW ^2^, g	2828	3080	91,0	0.096
Carcass weight, g	1751	1830	61,9	0.156
Total DMI ^3^, g/d	154	175	4,85	0.045
ADG ^4^, g/d	35	39	1,88	0.103
FCR ^5^, g DMI ^3^/g ADG ^4^	4.4	4.5	0.230	0.283
Tocopherols and Colesterol, µg/g muscle				
α-Tocopherol	2,19	1.92	0.111	0.127
Colesterol	1.24	0.97	0.098	0.101
Chemical composition				
Moisture	75.2	74.5	0.191	0.771
Crude protein	22.1	21.9	0.159	0.512
Ether extract	2.32	2.08	0.417	0.781
Ash	2.26	2.28	0.132	0.519

^1^ The treatments were as follows: only basal diet (C group) or the same basal diet in which part of cereals was replaced with 10% (DM on the diet fed) of coffee silverskin (CSS10 group). ^2^ BW = body weight; ^3^ DMI = dry matter intake; ^4^ ADG = average daily gain; ^5^ FCR = feed conversion ratio; ^6^ SEM= standard error of means.

**Table 3:** Effect of the dietary treatments on the fatty acid composition of *LTL* (g/100 g of fatty acids).

Item	Dietary Treatment		SEM	*p*-Value
	Control	CSS10		
intramuscular fat. mg/100 g of muscle	1623	1618	269	0.141
C10:0	3.40	2.71	0.775	0.956
C12:0	3.16	2.65	0.803	0.590
C14:0	31.6	36.7	6.580	0.315
C14:1 cis-9	1.20	2.76	0.437	0.085
C16:0	475	499	68.80	0.785
C16:1 cis-9	51.3	49.2	5.560	0.937
C17:0	11.4	14.6	2.480	0.568
C18:0	152	158	22.00	0.943
C18:1 cis-9	380	369	48.40	0.873
C18:2 cis-9. cis-12 LA ^1^	428	418	59.10	0.890
C18:3 n-3 ALA ^1^	27.1	18.7	3.020	0.089
C20:2 n-6	5.27	4.42	0.689	0.267
C20:3 n-6	9.33	5.26	1.750	0.097
C20:4 n-6	20.8	19,1	3.310	0.159
C20:5 n-3	1.86	0.84	0.318	0.064
C22:4 n-6	0.16	0.28	0.067	0.613
C22:5 n-3 DPA ^1^	10.19	4.25	1.640	0.069
C22:5 n-6	7.59	7.89	1.220	0.202
C22:6 n-3 DHA ^1^	2.39	2.16	0.453	0.157
C24:0	0.33	0.88	0.249	0.455
∑ SFA ^1^	677	716	101.0	0.797
∑ MUFA ^1^	433	421	55.90	0.864
∑ PUFA ^1^	512	481	66.80	0.709
∑ n-3	58.5	39.1	6.910	0.091
∑ n-6	463	447	59.80	0.775
n-6/n-3	7.91	11.4	0.752	0.035
Thrombogenic index ^2^	1.05	1.26	0.039	0.001
Atherogenic index ^3^	0.63	0.72	0.023	0.039

^1^ LA: linoleic acid; ALA: α-linolenic acid; DPA: docosapentaenoic acid; DHA: docosahexaenoic acid; SFA: saturated fatty acids; MUFA: monounsaturated fatty acids; PUFA: polyunsaturated fatty acids. ^2^ Thrombogenic index: (C14:0 + C16:0 + C18:0)/(0.5 MUFA + 0.5 PUFA n-6 + 3 PUFA n-3 + PUFA n-3/PUFA n-6). ^3^ Atherogenix index: (C12:0 + 4 × C14:0 + C16:0)/(MUFA + PUFA n-6 + PUFA n-3).

## Data Availability

The original contributions presented in the study are included in the article; further inquiries can be directed to the corresponding author.
